# Tumor DNA From Tumor *In Situ* Fluid Reveals Mutation Landscape of Minimal Residual Disease After Glioma Surgery and Risk of Early Recurrence

**DOI:** 10.3389/fonc.2021.742037

**Published:** 2021-10-11

**Authors:** Jinliang Yu, Zhiyuan Sheng, Shuang Wu, Yushuai Gao, Zhaoyue Yan, Chaojie Bu, Jianjun Gu, Yage Bu, Kaiyuan Deng, Sensen Xu, Zhongcan Chen, Qianqian Zhang, Ajmal Zemmar, Juha Hernesniemi, Meiyun Wang, Gang Liu, Tianxiao Li, Xingyao Bu

**Affiliations:** ^1^ Department of Neurosurgery, Children’s Hospital Affiliated to Zhengzhou University, Henan Children’s Hospital, Zhengzhou Children’s Hospital, Zhengzhou, China; ^2^ Department of Neurosurgery, Zhengzhou University People’s Hospital, Henan Provincial People’s Hospital, Zhengzhou, China; ^3^ Juha International Central Laboratory of Neurosurgery, Henan Provincial People’s Hospital, Zhengzhou, China; ^4^ Juha International Center for Neurosurgery, Henan Provincial People’s Hospital, Zhengzhou, China; ^5^ Department of Medical Imaging, Henan Provincial People’s Hospital, The People’s Hospital of Zhengzhou University, Zhengzhou, China; ^6^ Henan Provincial Key Laboratory for Imaging Diagnosis and Research of Neurological Diseases, Henan Provincial People’s Hospital, The People’s Hospital of Zhengzhou University, Zhengzhou, China; ^7^ Department of Center for Clinical Single Cell Biomedicine, Clinical Research Center, Department of Oncology, Henan Provincial People’s Hospital, The People’s Hospital of Zhengzhou University, Zhengzhou, China

**Keywords:** precision medicine, tumor *in-situ* fluid, circulating tumor DNA, spatiotemporal heterogeneity, glioma progression

## Abstract

The recurrence of glioma is a difficult problem in clinical treatment. The molecular markers of primary tumors after resection cannot fully represent the characteristics of recurrent tumors. Here, abundant tumor DNA was detected in tumor *in situ* fluid (TISF). We report that TISF-derived tumor DNA (TISF-DNA) can detect genomic changes in recurrent tumors and facilitate recurrence risk analysis, providing valuable information for diagnosis and prognosis. The tumor DNA in TISF is more representative and sensitive than that in cerebrospinal fluid. It reveals the mutational landscape of minimal residual disease after glioma surgery and the risk of early recurrence, contributing to the clinical management and clinical research of glioma patients.

## Introduction

Although the diagnosis and treatment of glioma has made great progress, the prognosis of patients is still not ideal (1). Almost all gliomas will recur after surgery. The recurrent glioma is evolved from the residual disease *in vivo* under natural and therapeutic pressure (2). Many studies have shown that there is a great difference between the primary tumor and the recurrent tumor (3–5). This complicates the development of effective treatment strategies and presents significant obstacles to the development of new targeted therapies (3). At present, molecular pathology obtained after resection of glioma has been used to guide postoperative treatment. However, due to the heterogeneity of recurrence and primary tumor, real-time postoperative gene status of glioma may be more accurate than that of tumor tissue in guiding postoperative treatment. Tumor circulating DNA (ctDNA) relapse is present in the early stage of tumor recurrence (6–8), but has not been confirmed in glioma. Real-time monitoring of residual disease progression after glioma resection and detection of ctDNA recurrence before imaging recurrence can realize clinical ultra-early treatment before recurrence. Detection of molecular characterization after recurrence of glioma *in vivo* can analyze the spatiotemporal heterogeneity of glioma, provide better clinical treatment strategies, and lay the foundation for breakthrough progress in clinical research.

Tumor DNA was extracted in the tumor *in situ* fluid (TISF) after glioma surgery. In our preliminary study, we reported that TISF, the fluid within the local surgical cavity of glioma, is a novel clinical source for real-time genomic profiling of glioma (9). Our results suggest that TISF-DNA can detect the genomic characteristics of early evolution of glioma after surgery and can characterize the genetic characteristics of recurrent gliomas, which may be more sensitive than CSF-ctDNA. It can monitor the clinical course of glioma recurrence in real time and provide guidance for early postoperative treatment and recurrence treatment.

## Materials and Methods

### Patients and Sample

**Figure 1:**
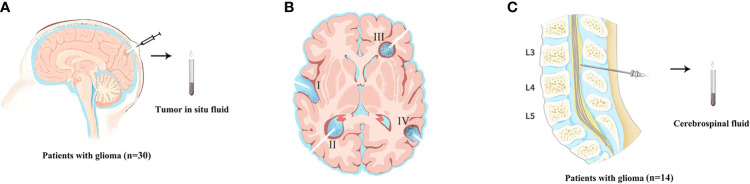
The sample acquisition of tumor DNA. **(A)** Tumor *in situ* fluid samples were obtained. (The state of the tumor at the time of TISF sampling. **(B-I/II)** TISF-1: sampled from early progression of residual disease. **(B-III/IV)** TISF-2: imaging showed tumor progression. TISF-a/TISF-b: Before MRI examination showed a recurrence of the tumor). **(C)** The sample of cerebrospinal fluid was obtained.

This retrospective cohort study was conducted on January 1, 2018, at the People’s Hospital of Henan Province on January 31, 2020. A total of 30 patients with brain glioma were diagnosed. A fluid reservoir sac (Medtronic, USA, **Supplementary Figure 1**) was placed during surgery and fixed between the periosteum and Galea aponeurosis for collection of TISF (**Figure 1**). Primary TISF samples from 30 patients were collected at two different postoperative times: The first time (TISF-1, **Figure 1B**_I/II_) is the 1 to 2 months after operation. The second time (TISF-2, **Figure 1B**_III/IV_) is that tumor progression was found during postoperative follow-up (according to RANO standard, T1 enhancement increased by ≥25%, T2/FLAIR increased, and new lesions and clinical manifestations deteriorated). Five patients received supplementary sampling before progression of the tumor. Cerebrospinal fluid samples were obtained from 14 patients at the time of tumor progression (**Figure 1C**). In addition, the matched blood samples were obtained from each patient, and a designed brain tumor map containing 68 genes was used to screen tumor mutation genes. The average depth of targeted sequencing of tumor tissue was 500X, the average depth of paired blood sequencing was 250X, and the average depth of TISF-DNA sequencing was 20,000X. Postoperative therapy was performed according to NCCN guidelines for central nervous system tumors. Fresh tumor tissue comes from surgical resection, and HE staining specimens contain more than 70% of tumor cells, which neuropathologists have confirmed. Grade III–IV glioma patients were followed up every 4–6 weeks, and grade II glioma patients were followed up every 2–3 months. All patients underwent MRI at each follow-up evaluation.

### Targeted Sequencing Analysis of Tumor-Associated DNA

All clinical TISF samples, CSF samples, and control tumor tissue samples were detected by Next-generation sequencing. QIAamp DNA Tissue and Blood Kit for Genomic DNA (Qiagen; Germantown, MD, USA) extract. TISF sample, CSF samples, and blood sample were centrifuged in EDTA tube at 1,900 g for 10 min, and the precipitate particles were frozen at −80°C. The supernatant was centrifuged at 16,000 g for 10 min and transferred to −80°C for preservation. CfDNA was extracted from TISF and blood supernatant using Mag-MAX CellFree DNA isolation kit (Thermo Fisher Scientific, Waltham, MA, USA). Finally, all segregated DNAs were quantified using the Qubit 2.0 Fluorometer with the Qubit dsDNA HS Assay kit (Life Technologies; Carlsbad, CA, USA).

As described elsewhere, the isolated DNA was cut into 150–200 bp fragments using Covaris M220 Focused-ultrasonicator™ Instrument (Covaris; Woburn, MA, USA). Following the manufacturer’s direction (10, 11), we constructed Fragmented DNA and ctDNA libraries with the KAPA HTP Library Preparation Kit (Illumina platforms; KAPA Biosystems; Wilmington, MA, USA). The DNA libraries were captured with a designed panel of 68 genes for brain tumors (GenetronHealth; Beijing, China), these containing major brain tumor-related genes. The DNA sequencing was based on novaseq high-throughput sequencing platform. After sequencing, we adopted such criteria that a mutation had an allele fraction of ≥0.1%, and a total of ≥4 reads were considered existing in liquid samples. Known recurrent loci were further manually checked with Integrative Genomics Viewer (IGV v2.3.34) in the target sample comparing to the normal blood DNA. The dbNSFP and the Exome Aggregation Consortium (ExAC) database were used to exclude either benign mutations with pp2_hdiv score <0.452 or polymorphic non-synonymous mutations sites. At the end, all detected mutations were annotated for genes using ANNOVAR, Oncotator and Vep.

### Statistical Analysis

We assessed differences in clinical characteristics between TISF-1-DNA and TISF-2-DNA patients using Fisher’s exact test for categorical variables and Wilcoxon test and Mann-Whitney (rank sum) test or Kruskal-Wallis test for continuous variables. Correlation between Tumor tissue Mutational Burden and TISF-DNA Number of mutations or TISF-DNA concentration was assessed by Spearman correlation. Multivariate analysis was performed using binary logistic regression analysis, and Hosmer-Lemeshow method was used to test the model fitting degree (*p* > 0.05 was highly fitting). We assessed the association between TISF-DNA detection and PFS and OS by the log-rank method. All statistical tests were two-sided, and *p* values < 0.05 were considered significant. Unless otherwise specified, SPSS (version 23.0; Armonk, NY, USA, IBM Corp) and GraphPad Prism (version 8.0c) were used for all analyses.

## Results

### Abundant Genomic Profiling of Glioma in TISF

**Figure 2:**
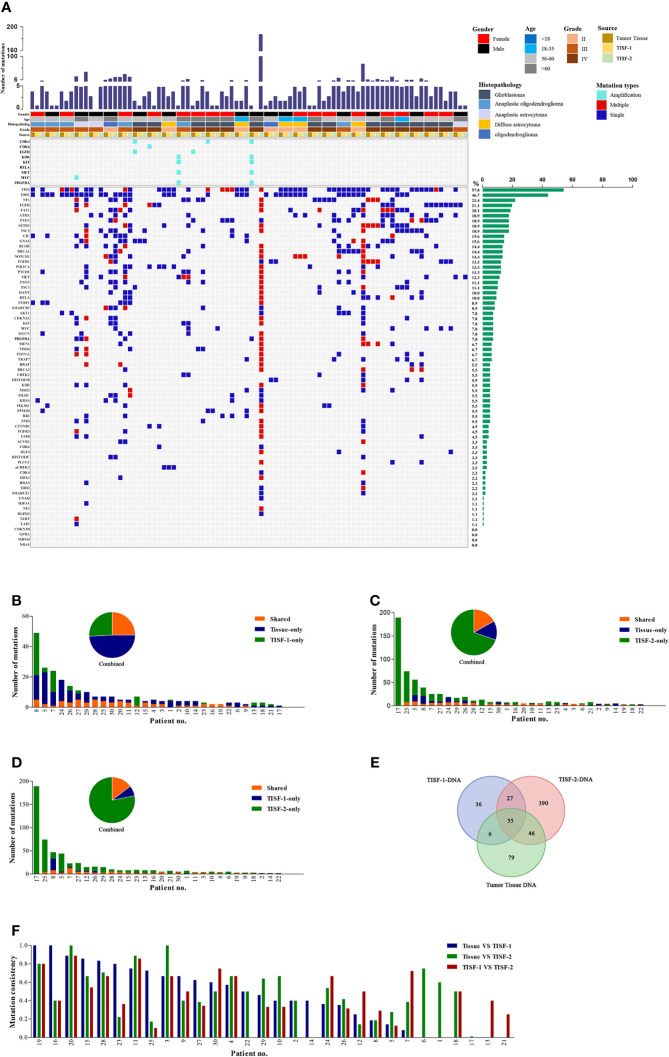
Mutant landscapes in tumor tissue and TISF. **(A)** Tumor DNA from the patient’s tumor *in situ* fluid was successfully isolated (n = 30). The following histologies were included in our study: glioblastoma, anaplastic oligoastrocytoma, anaplastic astrocytoma, diffuse astrocytoma, oligodendroglioma. The most frequently mutated genes included *IDH1*, *TP53*, *NF1*, *EGFR*, and *FAT1*. **(B-D)** Shared mutations in paired samples of tissue or TISF (n = 30). **(E)** Analysis of the frequency of mutation detected for three times. **(F)** Analysis of mutation consistency in pairs of TISF samples and matched tumor tissues.

We identified at least one tumor-derived TISF gene mutation from all 30/30 patients (100.0%) with tumor characteristics (**Table 1**), suggesting that TISF can be used to characterize recurrent gliomas. As shown in the addendum (**Figure 2A**), tumor tissue gene mutations were detected in 30 patients (30/30, 100.0%). There were 186 mutations, and the median of the mutation was 4. *TP53* (19/30, 63.3%) and *IDH1* (16/30, 53.3%) were the most common mutations. Abnormal gene rearrangement or copy number was detected in seven patients, and five cases occurred in glioblastoma (5/13, 38.5%). The most common is *CDK4* gene rearrangement or copy number abnormalities (4/7, 57.1%); all were found in glioblastoma.

**Table 1:** Clinical characteristics.

Variable	All (n = 32)
**Age, years**	
Median	53.2
Range	27-74
**Sex, n (%)**	
Female	16 (53.3)
Male	14 (46.7)
**Tumor grade (WHO), n (%)**	
II	7 (23.3)
III	9 (30.0)
IV	14 (46.7)
**Histopathology, n (%)**	
Glioblastoma, IDH-wild	14 (46.6)
Anaplastic oligodendroglioma, IDH-mutant	6 (20.0)
Anaplastic astrocytoma, IDH-mutant	2 (6.7)
Diffuse astrocytoma, IDH-mutant	6 (20.0)
Oligodendroglioma, IDH-mutant	2 (6.7)
**Aftertreatment, n (%)**	
Chemoradiotherapy	23 (76.7)
Chemotherapy	7 (23.3)
Location, n (%)	
Frontal lobe	9 (30.0)
Frontotemporal lobe	1 (3.3)
Temporal lobe	5 (16.7)
Temporoparietal lobe	5 (16.7)
Parietal lobe	3 (10.0)
Parietal-occipital lobe	3 (10.0)
Deep brain	3 (10.0)
Cerebellum	1 (3.3)

The TISF-1-DNA sequencing detected tumor gene mutations in 28 patients (93.3%, 28/30). The total number of mutations was 124, and the median was 2. The mutation rates of *TP53* (43.3%, 13/30) and *IDH1* (36.7%, 11/30) were still high, which was different from what we expected. In the TISF-2-DNA sequencing, the positive rate reached 100.0% (30/30), the number of mutations was 518, and the median was 6.5. *TP53* (19/30, 63.3%) and *IDH1* (14/30, 46.7%) had high mutation rates. In one case of diffuse astrocytoma, hereditary related gene mutation of ^a^ *CHEK2* was detected in three sequencings.

### Genomic Characteristics of Primary Glioma, Early Postoperative Tumor, and Recurrent Glioma

TISF-1 represents the genomic signature of early postoperative evolution of residual disease, and TISF-2 represents the genetic signature of tumor progression. We compared the detection results of tumor tissues and TISF-DNA detections (**Figures 2B–F**). In TISF and tumor samples, the percentage of shared mutations varies widely between samples (0–100%, **Figure 2F**), and the median total mutation rates were 43.1% (TISF-1) and 45.8% (TISF-2), respectively. In TISF-1 samples, the number of shared mutations with tumor tissue was 61, accounting for 25.0% (**Figures 2B, E**). In TISF-2 samples, there were more shared mutations (101, **Figure 2E**), but the proportion of shared mutations was significantly lower (16.9%, **Figure 2C**). The shared mutation of TISF-1 and TISF-2 was 82, and the median common mutation rate was 38.2%, accounting for 14.7% (**Figures 2D, E**).

**Figure 3:**
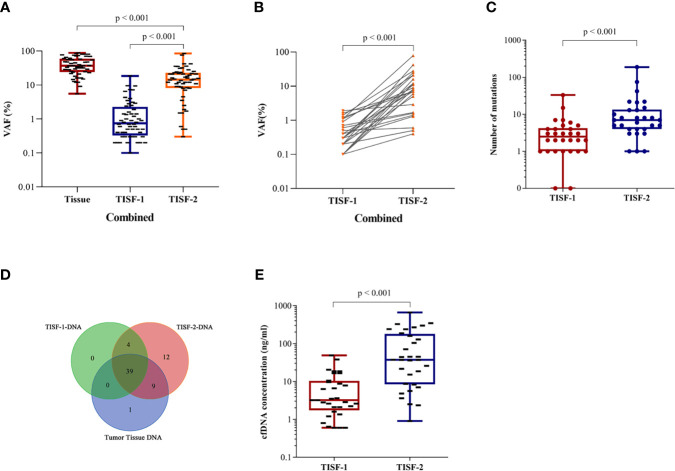
Longitudinal analysis of tumor DNA from TISF in different stages. **(A)** VAF changes in primary and recurrent tumor trunk mutations (*p* < 0.0001). **(B)** VAF of TISF shared mutation is elevated when tumor progression (*p* < 0.0001). **(C)** Elevated mutation load at tumor progression (*p* < 0.0001). **(D)** Twelve novel genotypes were detected in TISF-2 at tumor recurrence. **(E)** The concentration of TISF-cfDNA increased during tumor progression (*p* < 0.0001).

At the time of tumor recurrence, the variant allele fractions (VAFs) for TISF mutations were between 0.1 and 84.3%. For the shared trunk mutations of the primary tumor and the recurrent tumor, the increase of VAF was observed after the tumor was excised to the time of recurrence (**Figure 3A**, *p* < 0.0001). The shared mutations occurring in TISF also showed an increase in VAF (**Figure 3B**, *p* < 0.0001). We performed a longitudinal analysis of the dynamics of DNA mutation spectrum detected in 30 patients, and found that the total mutation load in the TISF-2-DNA spectrum increased when the tumor recurred (**Figure 3C**, *p * < 0.0001). When the tumor recurred, 24 patients developed new mutations that were not present in the original tumor. They contained common tumor-driven genes, such as *ATRX*, *PIK3CA*, *PTEN*, *NF1*, *RB1*, *SETD2*, *TP53* and other 12 mutation types (**Figure 3D**). At the same time, tumor recurrence was accompanied by the increase of TISF-cfDNA concentration (**Figure 3E**, *p* < 0.0001).

### TISF Is a More Sensitive Source of Glioma DNA Than the Cerebrospinal Fluid

**Figure 4:**
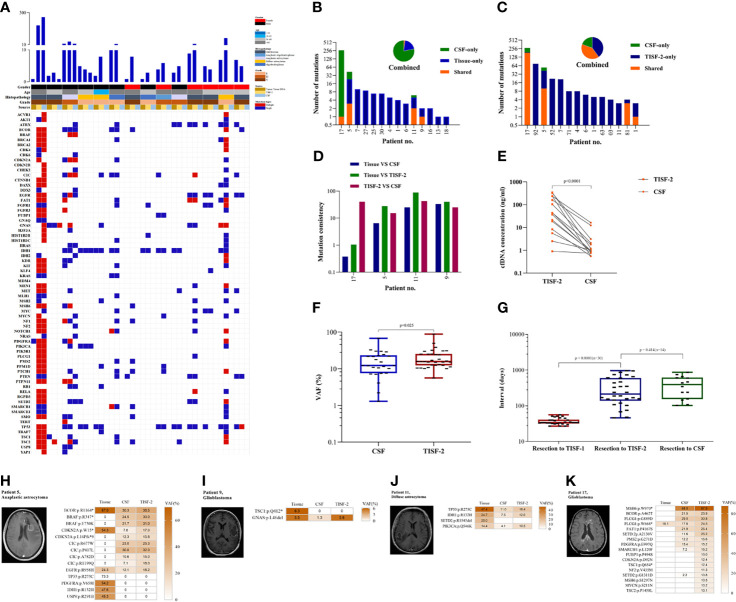
TISF is a more abundant source of tumor DNA. **(A)** The gene mutation profiles of the paired samples were in CSF and TISF. **(B, C)** Shared mutations in paired samples of CSF, tissue, or TISF (n = 14). **(D)** Mutation consistency analysis of CSF and matched tumor tissue and TISF samples. **(E)** cfDNA concentration in CSF and TISF-2. **(F)** CSF and TISF-2 mutated gene VAF. **(G)** The time interval between sample acquisition. **(H–K)** The four patients with positive CSF were found to have recurrent tumors with close communication with the ventricle and cistern, and the tumor burden was large. VAF of the trunk mutation was elevated at progression, but VAF was lower in CSF than in TISF.

In the cases matched with CSF, 100% (14/14) of tumor DNA was detected in TISF at the time of recurrence. In contrast, ctDNA was detected in only four patients (28.6%, 4/14) in CSF (**Figure 4A**). There were only one to three shared mutations in CSF with the primary tissue, and the results were similar to the mean of shared mutations in TISF-2 (mean 3.15), with a mutation consistency rate of 0.75–66.7% (**Figures 4B, D**). The shared mutations of CSF and TISF-2 have a high consistency (**Figure 4C**), with a consistency rate of 30.77–85.71% (**Figure 4D**), which also reflects the difference between the recurrence tumor and the primary tumor. Interestingly, we found a huge difference in cfDNA content between CSF and TISF samples. The concentration of cfDNA in TISF samples ranged from 0.9 to 346.5 ng/ml, while that in CSF was only 0.55–16.23 ng/ml (**Figure 4E**, *p* < 0.0001). The level of shared mutation VAF was higher than that of CSF in TISF (**Figure 4F**, *p* < 0.0001). There was no difference in the acquisition time of CSF and TISF samples (**Figure 4G**, p = 0.414). We found that the shared mutation VAF was lower than TISF in four patients who tested positive for CSF (**Figures 4H–K**).

Before the recurrence, 93.3% (28/30) of the patients were able to detect early evolution of glioma after surgery through TISF. Tumor DNA was found in 100.0% (30/30) of the patients at the time of tumor recurrence, while tumor DNA was found in only four patients (28.6%, 4/14) in CSF. These results are sufficient to indicate that TISF has higher tumor DNA content, higher detection positive rate, and higher clinical practicability.

### Tumor DNA in TISF and the Risk of Detection

**Figure 5:**
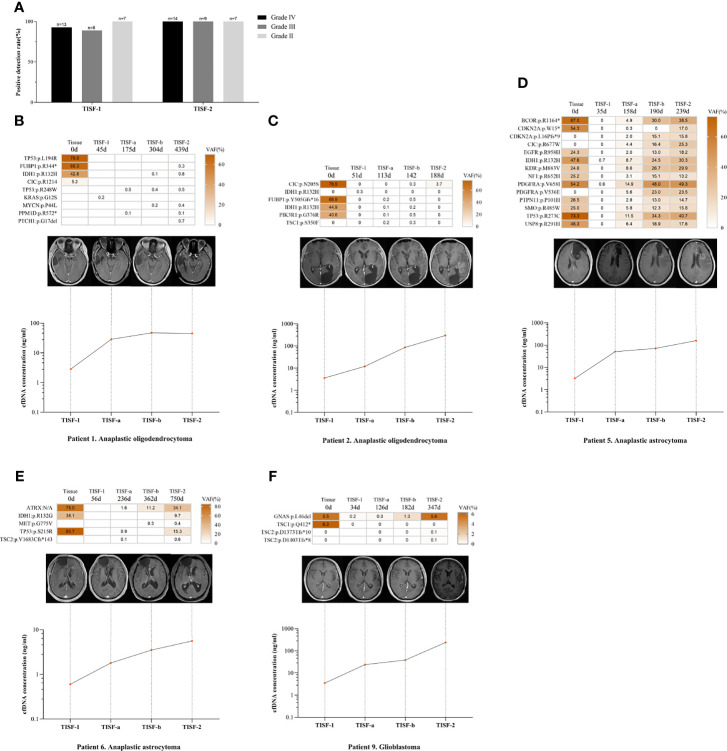
Relationship between tumor DNA derived from TISF and tumor progression. **(A)** Patients with different tumor grades detected positive for TISF. **(B, C)** Patients with increased cfDNA concentration and low VAF level when tumor progression. **(D–F)** The concentration of TISF-cfDNA and VAF continued to increase before imaging tumor progression.

To determine the risk factors for TISF tumor DNA detection, we compared TISF-DNA test results in patients grouped according to different clinicopathologic characteristics. Two positive TISF-DNA tests were not associated with tumor grade. Two patients without mutations in tumor tissue were removed. In TISF-1, the positive rate of TISF-DNA in grade IV patients was 92.8% (13/14), compared with 88.9% (8/9) in grade III patients and 100.0% (7/7) in grade II patients, and there was no difference in the positive rate of tumor DNA mutation (*p* = 1.000). In TISF-2, the positive rate of TISF-DNA was 100.0% (30/30). In contrast, there was no significant difference in the positive rate of different tumor grades (**Figure 5A**).

We compared the two TISF-DNA mutation loads of different grades of glioma. In TISF-1 and TISF-2, there was no difference in statistical results (*p* = 0.835, *p* = 0.575). Interestingly, it was found that the number of TISF-2-DNA mutations was higher than that of TISF-1-DNA in patients with grade IV and II (*p* = 0.001, *p* = 0.016). However, this difference was not found in patients with grade III (*p* = 0.161). The change of cfDNA concentration in TISF-1 and TISF-2 test samples was very significant; there was significant difference (*p* < 0.0001). TISF-1-cfDNA concentration was 0.60–48.50 ng/ml, and the median concentration was 3.20 ng/ml. The concentration of TISF-2-cfDNA was 0.90–346.00 ng/ml, and the median concentration was 37.25 ng/ml. We analyzed the changes of cfDNA concentration in TISF-1 and TISF-2 test samples of patients with different grades. The cfDNA concentrations of TISF-1-DNA and TISF-2-DNA in patients with grade IV, III, and II were significantly different (*p* = 0.001, *p* = 0.005, *p* = 0.012). The cfDNA concentration in TISF may be higher with the progression of tumor. The difference of cfDNA concentration in different tumor grades was significant. In TISF-1 (*p* = 0.007), the median cfDNA concentration was 7.20 ng/ml in grade IV, 3.02 ng/ml in grade III, and 1.07 ng/ml in grade II. In TISF-2 (*p* = 0.005), the median cfDNA concentration in patients with grade IV was 57.80 ng/ml. In contrast, the median cfDNA concentration in patients with grade III was 40.40 ng/ml, and that in patients with grade II was 5.95 ng/ml; there was significant difference.

We have found that the trunk mutation VAF is elevated in most gliomas with recurrence, but not in all. In patients we sampled multiple times (n=5, **Figures 5B–F**), patients 1 and 2 had lower levels of the shared mutated gene VAF when the tumor recurred (<5.0%), and patients 5, 6, and 9 showed a sustained increase in VAF. However, their levels of cfDNA concentration continued to rise as the tumor progressed, and no abnormal changes were found. 

### Higher cfDNA Concentration From TISF Sources Was Associated With Worse Progression-Free Survival

**Figure 6:**
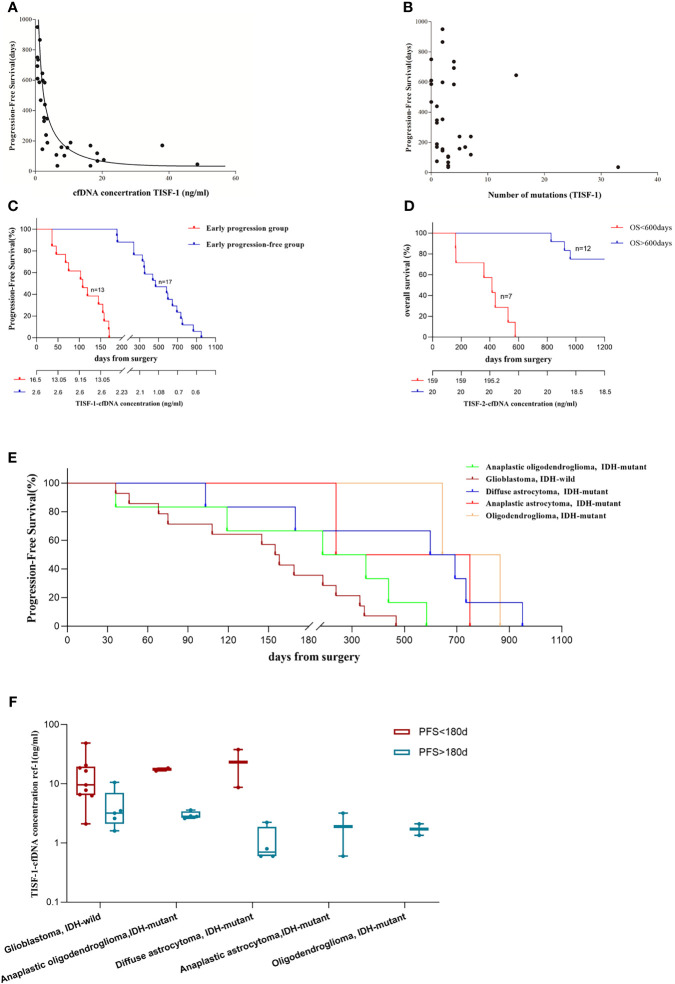
Analysis of tumor DNA derived from TISF and patient survival. **(A)** cfDNA concentration was inversely correlated with patients’ PFS (p < 0.001, Spearman’s rank correlation coefficient ρ= −0.844). **(B)** There was no correlation between the number of mutations in TISF-1 and patients’ PFS. **(C)** Progression-free survival in the early progression group and the early progression-free group. And the median cfDNA concentration at each time point. **(D)** Overall survival of 19 patients. **(E)** Progression-free survival of gliomas was observed for each pathological type. **(F)** For each pathological type of glioma, cfDNA concentration was still significantly higher in the early progression group.

TISF-1 represents genomic signatures for early postoperative evolution of glioma, while TISF-2 represents genomic signatures for tumor progression. We evaluated whether the early detection of TISF-DNA was related to the progression of glioma. We found that cfDNA concentration (TISF-1) was negatively correlated with PFS (*p* < 0.0001, Spearman’s rank correlation coefficient ρ = −0.844, **Figure 6A**), but the number of mutations in TISF-1 was not correlated with PFS (*p* = 0.242, **Figure 6B**). The patients were divided into early progression group (n = 13, grade IV 9, grade III 3, grade II 1) and early progression-free group (n = 17, grade IV 5, grade III 6, grade II 6) according to the presence or absence of the tumor progression within 180 days after surgery (**Figure 6C**). In the early progression group and early progression-free group, there was no difference in the number of mutations (*p* = 0.170, the median number of mutations were two and three). The high concentration of TISF-cfDNA in the early postoperative stage may mean patients at high risk of recurrence, because we found that the median cfDNA concentration in the early progressive group (16.5 ng/ml) was 6.3 times higher than that in the early non-progressive group (2.6 ng/ml) (*p* < 0.0001). The median PFS in the early progressive group and the early progression-free group was 108 days and 468 days, respectively.

In the multivariate analysis of this study, considering the patient’s age, tumor grade, tissue mutation number, TISF-1-DNA mutation number, and TISF-1-cfDNA concentration, it was found that the early postoperative cfDNA concentration was a high risk factor for worse PFS (<180 days, *p* = 0.026, OR = 1.638, 95% CL, 1.061–2.528, **Table 2**). The high concentration of cfDNA (TISF-2) at recurrence was also related to worse OS. Among the 19 patients who were followed up to OS (**Figure 6D**), the concentration of TISF-2-cfDNA (median concentration: 159.00 ng/ml) in patients with OS < 600 days was significantly higher than that in patients with OS > 600 days (median concentration: 20.00 ng/ml, *p* = 0.013). Even in each pathological type of glioma, TISF-1-cfDNA concentration was much higher in the early progression group than in the early progression-free group (**Figures 6E, F**).

**Table 2:** Multivariate analysis identified patients at high risk of worse PFS.

Variables	Univariate analysis		Multivariate analysis	
	OR (95% Cl)	P-value	OR (95% Cl)	P-value
Age (years)	1.049 (0.977–1.126)	0.185	0.982 (0.866–1.114)	0.781
Grade (II–IV)	3.363 (1.104–10.249)	0.033*	2.293 (0.440–11.955)	0.325
Number of mutations (tumor tissue)	1.011 (0.888–1.150)	0.874	0.910 (0.953–1.396)	0.666
Number of mutations (TISF-1-DNA)	1.083 (0.921–1.272)	0.335	1.177 (0.718–1.027)	0.518
cfDNA concentrations (TISF-1)	1.735 (1.139–2.643)	0.010*	1.638 (1.061–2.528)	0.026*

*Variables in the final equation; The logistic regression method: Enter; Hosmer-Lemeshow: χ^2 ^= 6.051, p = 0.641.

## Discussion

Residual disease after surgery is inevitable because of the diffuse growth of gliomas along blood vessels and white matter bundles, leading to almost all gliomas that eventually recur. At present, for glioma, molecular targeted therapy and early diagnosis of tumor DNA level recurrence are promising research projects, which are of great importance for the improvement of clinical treatment effect. Due to the special location of glioma, it is difficult to detect markers in the blood, and research in this field is quite limited at present. We found that TISF contains a large amount of tumor DNA, has a high positive rate (up to 93.3% positive at the early stage of tumor resection and 100% positive at tumor progression), and is even more sensitive than cerebrospinal fluid derived ctDNA, which can be used to characterize the genetic status of gliomas in real time. Due to the diffuse growth of glioma and the impossibility of 100% surgical resection, postoperative residual glioma is almost inevitable. The results of TISF-DNA detection in the early postoperative period showed that only 25% of the mutant genes were the same as those in the primary tissue, while only 16% of the mutant genes were the same as those in the primary tissue at the time of tumor recurrence, indicating the heterogeneity of recurrent tumors and primary tumors. At the time of tumor recurrence, increased VAF and mutation load were found in the common trunk mutation, and new mutated genes not present in the primary tumor were found in 24 patients. Many studies have found that circulating tumor DNA (ctDNA) is a reliable biomarker for residual tumor diseases and can be used to identify high-risk patients with tumor recurrence. These findings have been confirmed in tumors outside the nervous system, such as breast cancer (12), colon cancer (13), lung cancer (14, 15), esophageal cancer (16), and prostate cancer (17). But because of the existence of blood-brain barrier, the detection of ctDNA in blood is very limited. Although cerebrospinal fluid (CSF) may be a better source of ctDNA for glioma than blood (18–23), many related studies have shown that not all CSF can find ctDNA, the high negative rate is an undeniable fact, which brings difficulties to clinical application and dynamic follow-up research. The positive of CSF-ctDNA detection in brain tumors needs to meet the tumor progression, diffusion to the ventricle or subarachnoid space (20, 21, 24). This means that CSF-ctDNA is not a representative source of glioma ctDNA, and important information will be lost in the monitoring of tumor progression. In one study, the positive rate of CSF-ctDNA in 85 glioma patients receiving lumbar puncture after glioma resection was only 49.4% (20). In one study of medulloblastoma of the central nervous system, only 23.1% (3/13) of patients tested positive for CSF-ctDNA at postoperative follow-up (23). Wang Y et al. found that tumors adjacent to CSF or cortical surface are more common CSF-DNA mutations (25). Changcun Pan et al. found that tumors not directly adjacent to CSF cannot detect CSF-ctDNA mutation (19).

TISF is directly derived from possible postoperative residual tumor disease or tumor tissue that recurs *in situ*, so the use of TISF as a sample of glioma DNA is closest to the detection of tumor tissue itself. The spatial fluidity of local DNA in TISF is small, and it cleverly avoids the blood-brain barrier and the circulation of cerebrospinal fluid, so there is sufficient information of tumor DNA.

Only 28.6% (n = 4) of the 14 matched CSF-ctDNA cases were positive at the time of recurrence. The positive rate of TISF-DNA was 100% (30/30), and 93.3% (28/30) at the time of early recurrence. The content of cfDNA and the level of shared mutation VAF in TISF were higher than those in CSF, which indicated that the content of cfDNA in TISF-cfDNA from tumors was higher, and its sensitivity and effectiveness may be better than CSF-ctDNA. Early postoperative TISF level can reflect residual disease or early progress, which can be used as the baseline for subsequent detection. With the progression of the tumor, the gradual increase of VAF in the main mutation suggests the possibility of recurrence of the tumor, but the imaging may not show at this time, which helps to judge the recurrence of the tumor in advance to make clinical decisions. Early postoperative TISF-cfDNA concentration may represent residual disease or its early evolution and is related to worse PFS of patients, which is helpful to judge the prognosis of patients.

## Conclusions

Our study shows that the tumor DNA extracted from TISF can be used to characterize the genomic status of glioma in real time, which provides a novel avenue for glioma liquid biopsy. It may be more sensitive and representative than CSF-ctDNA. It helps to reveal the mutation landscape of minimal residual disease after glioma surgery and the risk of early recurrence, which is helpful for the clinical management and clinical research of glioma patients.

## Data Availability Statement

The data sets presented in this study can be found in online repositories. The names of the repository/repositories and accession number(s) can be found below: https://figshare.com/s/7ed5a16ca40d9a835f5b.

## Ethics Statement

The studies involving human participants were reviewed and approved by the Ethics Committee and Institutional Review Committee of the People’s Hospital of Henan Province. The patients/participants provided their written informed consent to participate in this study.

## Author Contributions

XB have full access to all data in the study and is responsible for the integrity of the data and the accuracy of the data analysis. JY and ZS contributed equally to this work. Concept and design: XB and JY. Acquisition, analysis, or interpretation of data: JY, ZS, YG, ZC, XB. Drafting of the manuscript: JY, ZS, XB. Critical revision of the manuscript for important intellectual content: All authors. Statistical analysis: JY, ZS, XB. Obtained funding: XB, TL. Administrative, technical, or material support: MW, JG. Supervision: XB. All authors contributed to the article and approved the submitted version.

## Funding

Science and Technology Tackle Program of Henan Province and Joint Project of Medical Science and Technology Tackling Plan of Henan Province (Grant nos. 192102310126 and 201601016). The project to improve the medical service capacity of provincial medical institutions (Grant no. CYQ20190073).

## Conflict of Interest

The authors declare that the research was conducted in the absence of any commercial or financial relationships that could be construed as a potential conflict of interest.

## Publisher’s Note

All claims expressed in this article are solely those of the authors and do not necessarily represent those of their affiliated organizations, or those of the publisher, the editors and the reviewers. Any product that may be evaluated in this article, or claim that may be made by its manufacturer, is not guaranteed or endorsed by the publisher.
