# Supplementary figures and images for: Tumor DNA From Tumor In Situ Fluid Reveals Mutation Landscape of Minimal Residual Disease After Glioma Surgery and Risk of Early Recurrence
Source: Front Oncol. 2021 Oct 11;11:742037. doi: 10.3389/fonc.2021.742037 (PMC8547270; doi:10.3389/fonc.2021.742037)

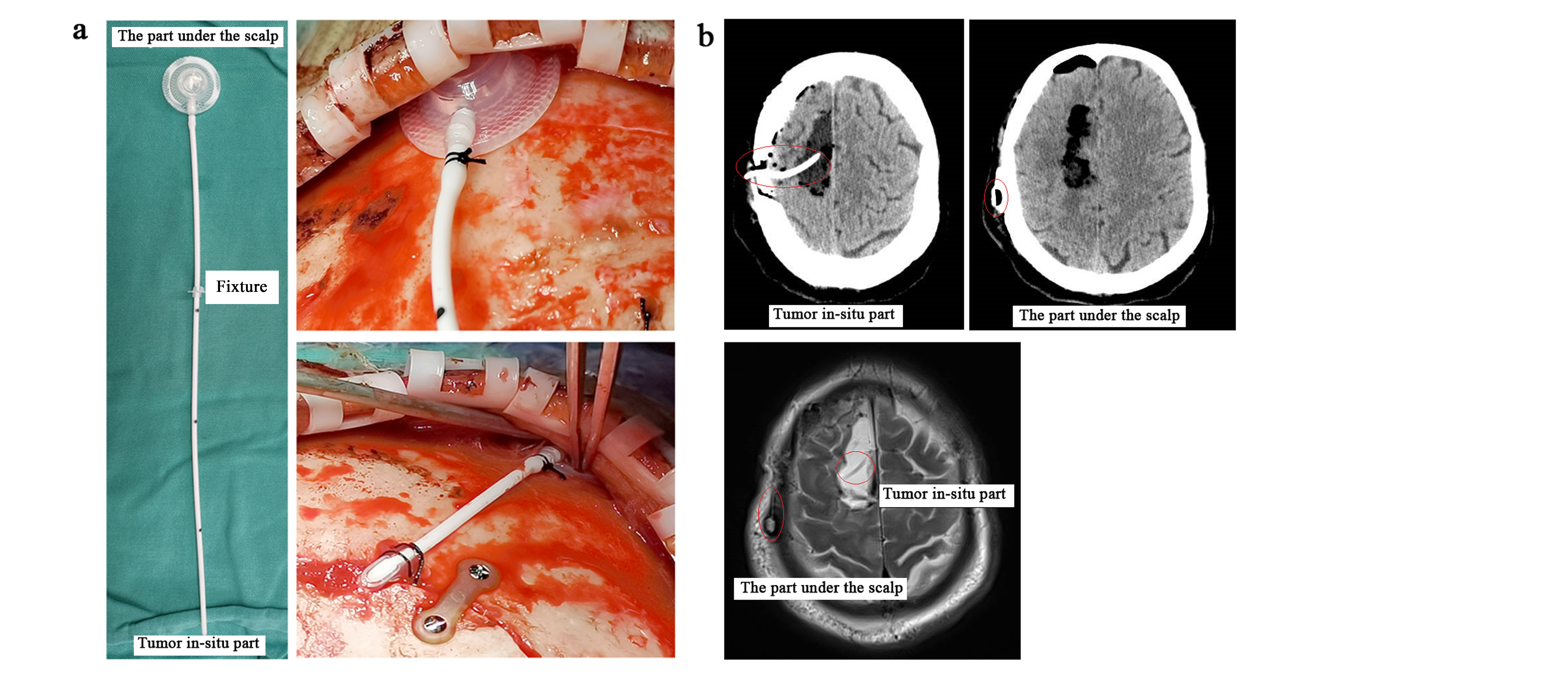

Supplement: Supplementary file 2 [file Image_1.tif]
